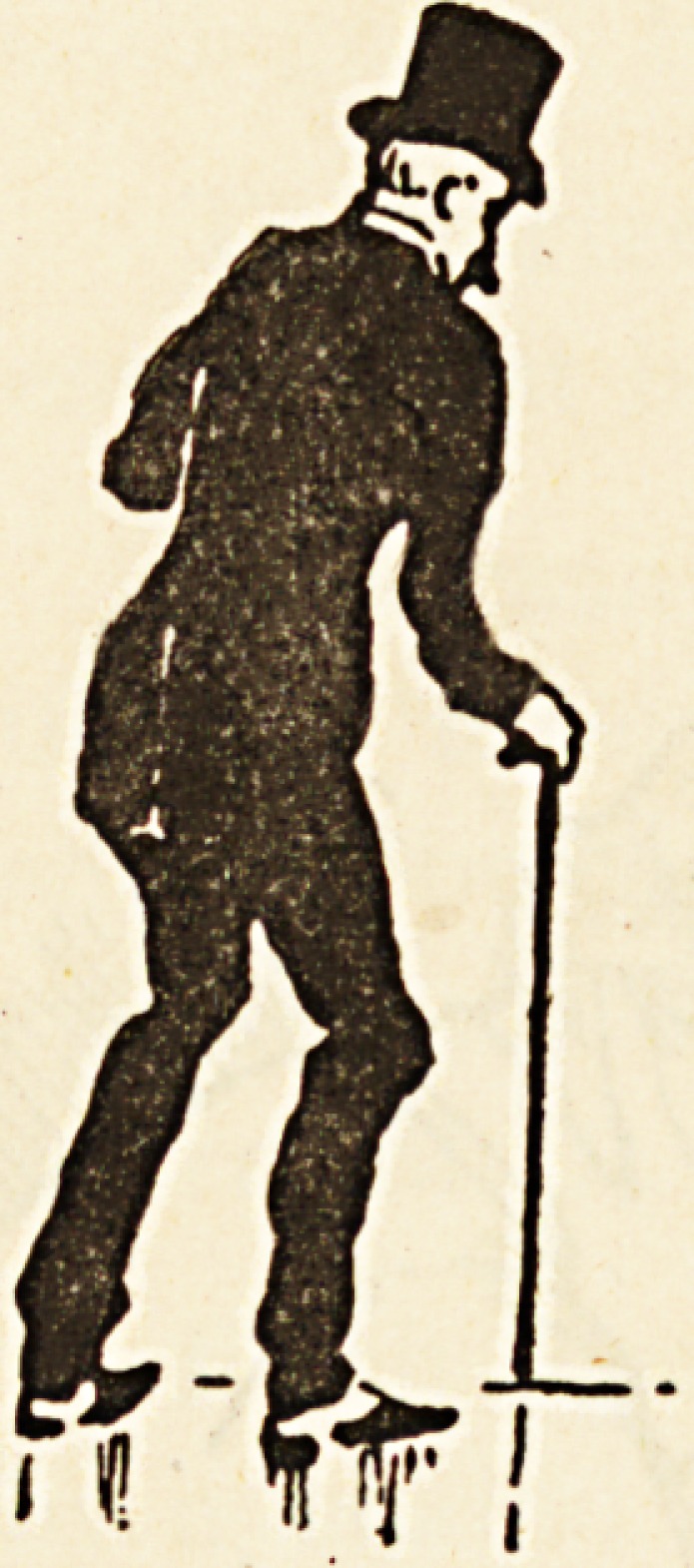# Scraps

**Published:** 1890-06

**Authors:** 


### SCRAPS

PICKED UP BY THE ASSISTANT-EDITOR.

**Out of Season.**—Le docteur B—, un de nos médicins légistes les plus éminents, a une antipathie passionnée, féroce, pour le piano. Dernièrement il dînait en ville. Après le dessert, concert improvisé. Un pianiste s'escrime avec ardeur. "L'abominable animal!" gronde le docteur à l'oreille de son voisin. "Que voulez-vous, cher ami, c'est son métier." "Son métier, belle raison! Est-ce que je vais faire des autopsies dans les salons, moi?"—*L'Union médicale.*

**The Doctor and his Journal.**—We believe that a first duty of the medical man is to help develop, foster, and sustain the medical societies and medical interest of his own locality. He has power personally to stimulate his associates, and to aid them in organization and in medical progress. In the development of such local interests, nothing can be so helpful as the ably-conducted and well-supported local medical journal. To this he owes a primary obligation, both literary and pecuniary. Its pages should be replete with the recorded experiences of local contributors; while, in turn, it should garner for them the best of medical productions from all lands.—*The Journal of the American Medical Association.*

**Imaginary Illness.**—Nothing more amusing has been written on this subject than the following by Mr. Jerome K. Jerome in his clever *Three Men in a Boat*:

"I had just been reading a patent liver-pill circular, in which were detailed the various symptoms by which a man could tell when his liver was out of order. I had them all.

"It is a most extraordinary thing, but I never read a patent medicine advertisement without being impelled to the conclusion that I am suffering from the particular disease therein dealt with in its most virulent form. The diagnosis seems in every case to correspond exactly with all the sensations that I have ever felt. I remember going to the British Museum one day to read up the treatment for some slight ailment of which I had a touch—hay fever, I fancy it was. I got down the book, and read all I came to read; and then, in an unthinking moment, I idly turned the leaves, and began to indolently study diseases generally. I forget which was the first distemper I plunged into—some fearful, devastating scourge, I know—and, before I had glanced half down the list of 'premonitory symptoms,' it was borne in upon me that I had fairly got it.

"I sat for awhile, frozen with horror; and then in the listlessness of despair, I again turned over the pages. I came to typhoid fever—read the symptoms—discovered that I had typhoid fever, must have had it for months without knowing it—wondered what else I had got; turned up St. Vitus's Dance—found, as I expected, that I had that too,—began to get interested in my case, and determined to sift it to the bottom, and so started alphabetically—read up ague, and learnt that I was sickening for it, and that the acute stage would commence in about another fortnight. Bright's disease, I was relieved to find, I had only in a modified form, and, so far as that was concerned, I might live for years. Cholera I had, with severe complications; and diphtheria I seemed to have been born with. I plodded conscientiously through the twenty-six letters, and the only malady I could conclude I had not got was housemaid's knee.

"I felt rather hurt about this at first; it seemed somehow to be a sort of slight. Why hadn't I got housemaid's knee? Why this invidious reservation? After a while, however, less grasping feelings prevailed. I reflected that I had every other known malady in the pharmacology (*sic*), and I grew less selfish, and determined to do without housemaid's knee. Gout, in its most malignant stage, it would appear, had seized me without my being aware of it; and zymosis I had evidently been suffering with from boyhood. There were no more diseases after zymosis, so I concluded there was nothing else the matter with me.

"I sat and pondered. I thought what an interesting case I must be from a medical point of view, what an acquisition I should be to a class! Students would have no need 'to walk the hospitals,' if they had me. I was a hospital in myself. All they need do would be to walk round me, and, after that, take their diploma.

"Then I wondered how long I had to live. I tried to examine myself. I felt my pulse. I could not at first feel any pulse at all. Then, all of a sudden, it seemed to start off. I pulled out my watch and timed it. I made it a hundred and forty-seven to the minute. I tried to feel my heart. I could not feel my heart. It had stopped beating. I have since been induced to come to the opinion that it must have been there all the time, and must have been beating, but I cannot account for it. I patted myself all over my front, from what I call my waist up to my head, and I went a bit round each side, and a little way up the back. But I could not feel or hear anything. I tried to look at my tongue. I stuck it out as far as ever it would go, and I shut one eye, and tried to examine it with the other. I could only see the tip, and the only thing that I could gain from that was to feel more certain than before that I had scarlet fever.

"I had walked into that reading-room a happy, healthy man. I crawled out a decrepit wreck.

"I went to my medical man. He is an old chum of mine, and feels my pulse, and looks at my tongue, and talks about the weather, all for nothing, when I fancy I'm ill; so I thought I would do him a good turn by going to him now. 'What a doctor wants,' I said, 'is practice. He shall have me. He will get more practice out of me than out of seventeen hundred of your ordinary, commonplace patients, with only one or two diseases each.'

"Going back to the liver-pill circular, I had the symptoms, beyond all mistake, the chief among them being 'a general disinclination to work of any kind.' What I suffer in that way no tongue can tell. From my earliest infancy I have been a martyr to it. As a boy, the disease hardly ever left me for a day. They did not know, then, that it was my liver. Medical science was in a far less advanced state than now, and they used to put it down to laziness."

## Figures and Tables

**Figure f1:**
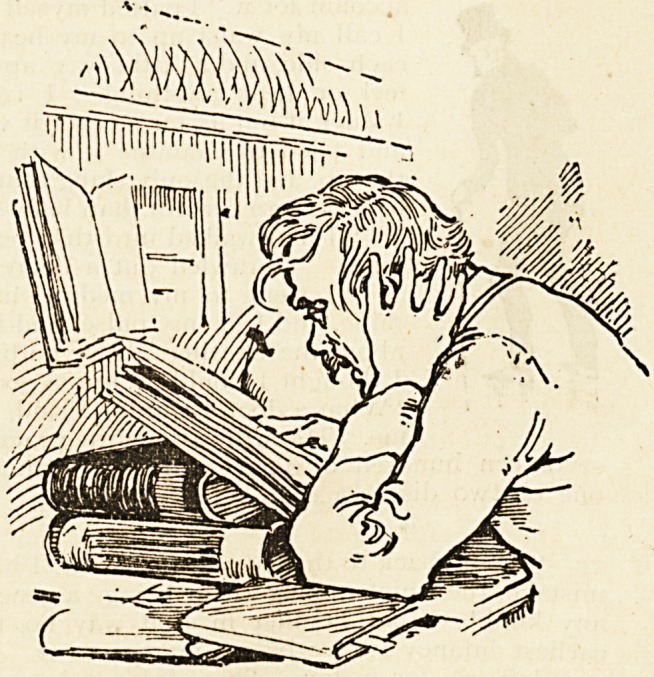


**Figure f2:**